# Draft Genome Sequence of *Pseudoalteromonas* sp. Strain ECSMB14103, Isolated from the East China Sea

**DOI:** 10.1128/genomeA.00330-15

**Published:** 2015-04-23

**Authors:** Xing-Pan Guo, De-Wen Ding, Wei-Yang Bao, Jin-Long Yang

**Affiliations:** a Key Laboratory of Exploration and Utilization of Aquatic Genetic Resources, Shanghai Ocean University, Ministry of Education, Shanghai, China; b Marine Ecology Research Center, First Institute of Oceanography, State Oceanic Administration, Qingdao, China; c Institute of Marine Science and Technology, Yangzhou University, Yangzhou, China

## Abstract

*Pseudoalteromonas* sp. strain ECSMB14103 was isolated from marine biofilms formed on the East China Sea. The draft genome sequence comprises 4.11 Mp with a G+C content of 39.7%. The information from the draft genome will contribute to an understanding of bacteria-animal interaction.

## GENOME ANNOUNCEMENT

*Pseudoalteromonas* is a genus of *Gammaproteobacteria* that is widespread in the oceans of the world, from surface water to deep-sea sediments (1). Many *Pseudoalteromonas* species are frequently found in association with eukaryotic hosts in the marine environment and produce biologically active molecules (2). Interest in the formation of biofilms of *Pseudoalteromonas*, microbe-host interaction, and the synthesis of molecules has been increasing since 1995. Recent research of the literature from the last 20 years on the topic “*Pseudoalteromonas*” yielded more than 1,400 references, and more than 1,000 of the references are from the last 10 years. *Pseudoalteromonas* species could affect the process of larval settlement and metamorphosis of many marine invertebrates, such as coral (3), bryozoan (4), polychete (5), mussel (6), barnacle (7), and sea urchin (8). Recently, some species of *Pseudoalteromonas* have also been known to promote the settlement of juveniles of the mussel *Mytilus coruscus* (9), an important fouling and aquaculture species in the East China Sea. Here, we present the genome sequence of *Pseudoalteromonas* sp. ECSMB14103 for the purpose of elucidating its particular molecular cues and mechanisms that promote the recruitment of larvae and juveniles of the mussel *M. coruscus*.

*Pseudoalteromonas* sp. strain ECSMB14103 was isolated from marine biofilms developed on glass slides immersed at a depth of 0.5 to 1.0 m below the surface of the East China Sea (122°46′ E, 30°43′ N), and the 16S rDNA sequences of *Pseudoalteromonas* sp. ECSMB14103 shared 99% similarity with *Pseudoalteromonas marina* Mano4 (accession no. AY563031) (6). The draft genome sequences of the strain ECSMB14103 were obtained by the Shanghai Majorbio Pharm Technology Co., Ltd., (Shanghai, China) using the Illumina MiSeq platform with a paired-end library. After being trimmed and merged, the reads were *de novo* assembled with the GS De Novo assembler version 2.8. Open reading frames (ORFs) were predicted by using the Glimmer version 3.02 program (10). All ORFs were then annotated by comparison with the NCBI-NR and KEGG databases using BLASTp (BLAST 2/2/28+). The tRNA and rRNA were predicted by the tRNAscan-SE version 1.3.1 (11) and Barrnap version 0.4.2 (http://www.vicbioinformatics.com/software.barrnap.shtml) programs, respectively.

The draft genome sequence of the ECSMB14103 strain comprises 4.11 Mb, which is assembled into 32 contigs, with the size of the largest contig being 1,079,592 bp. The *N*_50_ and *N*_90_ quality measurements of the contigs were 413,937 bp and 117,763 bp, respectively. The G+C content is 39.7%. The genome contains 3,675 predicted protein-coding sequences, 121 tRNA genes for 20 amino acids, and 4 rRNA genes. The availability of the genome sequence of *Pseudoalteromonas* sp. ECSMB14103 can provide insight into exploring the molecular cues involved in the recruitment of the mussel *M. coruscus* larvae and juveniles.

### Nucleotide sequence accession numbers. 

This whole-genome shotgun project has been deposited at DDBJ/EMBL/GenBank under the accession number JWGY00000000. The version described in this paper is the first version, JWGY01000000.
